# Cardiovascular Disease in Type 1 Diabetes Mellitus: Epidemiology and Management of Cardiovascular Risk

**DOI:** 10.3390/jcm10081798

**Published:** 2021-04-20

**Authors:** Cristina Colom, Anna Rull, José Luis Sanchez-Quesada, Antonio Pérez

**Affiliations:** 1 Endocrinology and Nutrition Department, Hospital de la Santa Creu i Sant Pau-Hospital Dos de Maig, 08041 Barcelona, Spain; cristinacolomcomi@gmail.com; 2 Cardiovascular Biochemistry Group, Research Institute of the Hospital de la Santa Creu i Sant Pau (IIB Sant Pau), 08041 Barcelona, Spain; anna.rull@iispv.cat (A.R.); jsanchezq@santpau.cat (J.L.S.-Q.); 3 Hospital Universitari Joan XXIII, IISPV, Universitat Rovira i Virgili, 43002 Tarragona, Spain; 4 CIBER of Diabetes and Metabolic Diseases (CIBERDEM), 08041 Barcelona, Spain; 5 Department of Medicine, Universitat Autonoma de Barcelona (UAB), 08193 Barcelona, Spain

**Keywords:** type 1 diabetes, cardiovascular disease, prevalence, risk factor, lipoproteins, hyperglycemia, risk stratification, therapy, management

## Abstract

Cardiovascular disease (CVD) is a major cause of mortality in type 1 diabetes mellitus (T1DM) patients, and cardiovascular risk (CVR) remains high even in T1DM patients with good metabolic control. The underlying mechanisms remain poorly understood and known risk factors seem to operate differently in T1DM and type 2 diabetes mellitus (T2DM) patients. However, evidence of cardiovascular risk assessment and management in T1DM patients often is extrapolated from studies on T2DM patients or the general population. In this review, we examine the existing literature about the prevalence of clinical and subclinical CVD, as well as current knowledge about potential risk factors involved in the development and progression of atherosclerosis in T1DM patients. We also discuss current approaches to the stratification and therapeutic management of CVR in T1DM patients. Chronic hyperglycemia plays an important role, but it is likely that other potential factors are involved in increased atherosclerosis and CVD in T1DM patients. Evidence on the estimation of 10-year and lifetime risk of CVD, as well as the efficiency and age at which current cardiovascular medications should be initiated in young T1DM patients, is very limited and clearly insufficient to establish evidence-based therapeutic approaches to CVD management.

## 1. Introduction

The prevalence of diagnosed type 1 diabetes (T1DM) among US adults in 2016 and 2017 was 0.5% [1] and is increasing worldwide, which may be partly related to reduced natural selection associated with health advances [2]. Over the past few decades, reduction in cardiovascular mortality and coronary cardiovascular disease in non-diabetic and diabetic patients has corresponded with an overall mortality reduction and significant improvement in life expectancy in patients with type 2 (T2DM) and type 1 diabetes mellitus (T1DM) [3,4]. These findings are reflected clearly in the two cohorts of the Pittsburgh Epidemiology of Diabetes Complication (EDC) Study, which found an increase of 14 years in life expectancy between T1DM patients diagnosed in 1950–1964 and those diagnosed in 1965–1980 [5,6]. This improvement seems to be related to the optimization of glycemic control, management of cardiovascular risk (CVR) factors, and interventional cardiology. However, the total risk of cardiovascular disease (CVD) remains higher in T1DM patients than in non-diabetic subjects, particularly in women, and it becomes the most important cause of morbidity and mortality in T1DM patients [7,8,9,10]. Compared to early-onset T2DM, T1DM is associated with a lower mortality and fewer complications [11,12]. The physiopathology underlying the relationship between cardiovascular events and T1DM is poorly understood, and assessment of CVR and management to reduce CVD has been extrapolated in part from studies conducted in T2DM patients and still represents a challenge for clinicians. The present article reviews epidemiological studies that demonstrate CVD’s relevance in T1DM patients, current knowledge about risk factors involved in the relationship between T1DM and CVD, and factors relevant to the evaluation and management of T1DM. Also, we discuss evidence that stratifies CVR and addresses CVR factors, and we summarize current clinical practice recommendations on preventing, or at least delaying, CVD in T1DM patients. 

## 2. Epidemiology of Cardiovascular Disease in T1DM Patients

### 2.1. Clinical Atherosclerosis in T1DM Patients

The main epidemiological studies demonstrate that CVD events are more common and occur earlier in T1DM patients than in the general population, although CVD prevalence varies substantially depending on diabetes duration, age, and gender (Table 1). Krolewski et al. in 1987 [13], published the first observational study that included cumulative mortality rate (CMR) due to coronary artery disease (CAD) in T1DM patients. By 55 years of age, the total CMR due to CAD was 35 ± 5%, much higher than the corresponding rate for non-diabetic subjects in the Framingham Heart Study, which was 8% for men and 4% for women. Also, the combined CMR for clinical coronary heart disease (CHD), including angina and acute nonfatal myocardial infarction, and asymptomatic CAD detected by stress test was 33% among T1DM survivors ages 45–59. 

Since then, many studies progressively have confirmed high CVD prevalence in T1DM patients compared with the overall population. In the Wisconsin Epidemiologic Study of Diabetic Retinopathy (WESDR), the standardized mortality ratio (SMR) for ischemic heart disease in a diabetic group (*n* = 1200) diagnosed before age 30 and taking insulin was 9.1 in men and 13 in women [14]. The Pittsburgh Epidemiology of Diabetes Complications (EDC) study demonstrated that CAD events were the leading cause of death in T1DM patients. The incidence of major CAD events in T1DM adults ages 28–38 was 0.98% per year and surpassed 3% per year after age 55 [15], with SMR ratios of CVD at 8.8 and 24.7 for men and women, respectively, in the Allegheny County Type 1 Diabetes Registry [9]. In the EURODIAB IDDM Complications Study, which included 3250 T1DM patients from 16 European countries, overall CVD prevalence was 9% in men and 10% in women. In addition, CVD prevalence increased with diabetes mellitus (DM) duration and age, at 6% in T1DM patients ages 15–29, and 25% in T1DM patients age 45–59 [16]. A study of the CVD prevalence rate was conducted on T1DM patients who were selected to be comparable in age (mean age of 28) and DM duration (18–20 years) to the EDC Study and the EURODIAB IDDM Complications Study [17]. This report confirms the high prevalence of CVD in TIDM subjects and was similar in both populations, (i.e., men 8.6% vs. 8.0%, women 7.4% vs. 8.5%, EURODIAB vs. EDC, respectively). The UK General Practice Research Database (GPRD), one of the most robust analyses of CVD risk that includes data from more than 7400 T1DM patients with a mean age of 33 ± 14.5 years and mean DM duration of 15 ± 12 years, reported that CVD events occurred about 10 to 15 years earlier in T1DM patients than in the matched non-diabetic control group. During a mean follow-up of 4.7 years, the hazard ratio (HR) for major CVD events was 3.6 and 7.7 in T1DM men and women, respectively, after stratification by year of birth and gender [18,19]. 

Another comparative study, published by the Diabetes Control and Complications Trial/Epidemiology of Diabetes Interventions and Complications (DCCT/EDIC) Research Group and Collaborators, comprises an analysis of the long-term cumulative incidence of CVD events in T1DM patients [20]. Either intensive or conventional therapy patients in the DCCT/EDIC study cohort (*n* = 1441) were compared with a similar subset of the EDC population (*n* = 161) after 18.5 years of follow-up. The cumulative incidence of CVD in the DCCT/EDIC conventional treatment group was similar to the EDC cohort, with 14% cumulative incidence, but was significantly higher than the 9% of the DCCT/EDIC intensive treatment group. These findings reflect that the frequency of acute complications in T1DM patients, especially for those under intensive therapy over time, was lower than that previously published. 

Recently, some observational studies reported that although the situation has improved for T1DM patients over the past few years, cardiovascular event rates and cardiovascular mortality rates for CVD remain higher in T1DM patients than in the overall population [7,21,22]. A registry-based observational study conducted in a Swedish population of 34,000 T1DM patients reported a higher risk of total death and CVD death rates––two- to nine-fold times and three- to 10-fold times, respectively––in T1DM patients than in matched controls, depending on glycated hemoglobin (HbA1c) levels [22]. In the Scottish Care Information-Diabetes Collaboration (SCI-DC) database, the age-adjusted incidence rate ratio (IRR) for a first CVD event associated with T1DM (*n* = 21,789) vs. the non-diabetic population (3.96 million) was higher in women (3.0: 95% CI 2.4–3.8) than in men (2.3: 2.0–2.7), while the IRR for all-cause mortality associated with T1DM was comparable at 2.6 (2.2–3.0) in men and 2.7 (2.2–3.4) in women [21]. The estimated loss of life expectancy for patients with type 1 diabetes in Scotland at age 20 was approximately 11 years for men and 13 years for women compared with the general population without T1DM [7]. Finally, in the Swedish National Diabetes Registry, T1DM patients with disease onset before age 10 experienced a 30-fold increased risk of CHD and acute myocardial infarction (AMI) in their early adult years, and women displayed a 60- and 90-fold increased risk of CHD and AMI, respectively. Early onset type 1 diabetes resulted in a loss of 17.7 and 14.2 years of life expectancy for women and men, respectively [8]. 

In conclusion, CVD incidence is much higher and happens earlier in T1DM patients, women are more affected than men, and the incidence is much more pronounced in patients with early diabetes. In T1DM, relative CVD risk, age-adjusted, was 4- to 10-fold times higher, and CVD events occurred about 10 to 15 years earlier than matched non-diabetic subjects. CVD prevalence depends on DM duration, glycemic control, and age of patients included in the cohorts. 

**Table 1:** Main epidemiological studies revealing increased cardiovascular disease (CVD) risk and mortality in type 1 diabetes mellitus (T1DM) patients.

Study	Population	Comparison Population	Follow Up (Years)	CVD Events	CVD Mortality
Observational study of T1DM (Joslin Diabetes Center) [13]	292 newly diagnosed T1DM patients	No comparison group (NCG)	20–40	Cumulative mortality rate (CMR) for coronary heart disease (CHD): 33% among survivors ages 45–59	CMR due to coronary artery disease (CAD): 35%
Wisconsin Epidemiologic Study of Diabetic Retinopathy (WESDR) [14]	1200 younger onset diabetic patients; 1772 older onset diabetic patients	General Wisconsin population	8.5		Younger onset: standardized mortality rate (SMR) (95% CI)—ischemic heart disease (IHD): 9.1 (5.9–13.4) men (M), 13.5 (6.7–24.2) women (W) and cerebrovascular disease: 4.1 (0.8–11.8) (M + W) Older onset: SMR (95% CI) IHD 4.4 (2.1–2.8) (M); 2.2 (1.9–2.6)(F) and cerebrovascular disease: 2.0 (1.6–2.5) (M + W)
Pittsburgh Epidemiology of Diabetes Complications (EDC) [9]	1075 T1DM patients diagnosed from 1965–1979 (age: 42.8 ± 8.0 years; diabetes duration: 32.0 ± 7.6 years)	Age-, sex-, and race-matched general population	Cross-sectional study		SMR (95% CI) for CVD: 8.8 (6.3–11.2) (M) 24.7 (17.9–31.6) (W)
EURODIAB IDDM Complications study [16]	3250 T1DM patients (ages: 33 ± 10 years; diabetes duration: 14.7 ± 9.3 years)	NCG	Cross-sectional study	CVD prevalence: 9% (M) 10% (W)	
EDC Study compared with the EURODIAB IDDM Complications Study [17]	-EDC: 286 male patients (diabetes duration: 20.1 years) and 281 female patients (diabetes duration: 19.9 years); -	EURODIAB 608 M patients (diabetes duration: 18.1 years) and 607 W patients (diabetes duration: 18.9 years)	Cross-sectional comparison study	CVD Prevalence: EDC: 8% (M), 8,5% (W) EURODIAB: 8.6% (M) 7.4% (W)	
UK General Practice Research Database (GPRD) [18]	7479 T1DM patients; 38,116 non-DM patients	38,116 controls subjects without diabetes	From 1992 to 1999 (mean 4.5 ± 2.26 years)	HR (95% CI): 3.6 (2.9–4.5) (M) HR (95% CI) 7.7 (5.5–10.7) (W)	CVD mortality HR (95% CI): 5.8 (3.9–8.6) (M) CVD mortality HR (95% CI): 11.6 (6.7–20, 1) (W)
DCCT/EDIC cohort and a subset of the EDC cohort [20]	DCCT/EDIT: 1441 T1DM patients; EDC: 161 T1DM patients		Study after a diabetes duration of 30 years	Cumulative incidence: DCCT/EDIC- Conventional therapy: 14% EDC cohort: 14% DCCT/EDIC intensive therapy: 9%	
Swedish National Diabetes Register [22]	33,915 T1DM patients;	169,249 controls subjects without diabetes	8 years		CVD mortality: HR (95% CI), 4.60 (3.47–6.10); Total mortality: HR (95% CI), 3.52 (3.06–4.04)
Scottish Registry Linkage Study [21]	21,789 T1DM patients; 3.96 million non-DM patients	Non diabetic Scottish population (3.96 million)	Cross-sectional comparison study (Nationwide, register-based cohort study)	incidence rate ratio (IRR) for first CVD (95% CI): 2.3 (4.0–2.7) (M) 3.02 (2.4–3.8) (W)	IRR (95% CI) for all-cause mortality: 2.6 (2.2–3) (M) 2.7 (2.2–3.4) (W)
Meta-analysis [10]	26 studies (214,114 individuals) published between 1996 and 2014)				SMR (95% CI) for CVD T1DM vs. non-DM 11.30 (6.87–18.59) in W and 5.68 (3.82–8.44) in M
Allegheny County type 1 diabetes Registry [23]	1075 childhood-onset T1DM patients, 1965–1979	General Allegheny County population	33.0 years		Total mortality SMR (95% CI): 5.0 (4.0–6.0) (M) 13.2 (10.7–15.7) (W)
Norwegian Childhood Diabetes Registry [24]	1906 childhood-onset T1DM patients diagnosed between 1973 and 1982	General Norway population	24.2 ± 3.9 years		CVD mortality SMR (95% CI): 11 (5.2–18.8) (M); 10.3 (2.7–22.7) (W) IHD mortality SMR (95% CI): 20.2 (7.3–39.8) (M) 20.6 (1.8–54.1) (W)
Finnish Diabetic Nephropathy Study (FinnDiane) cohort [25]	10,737 children newly diagnosed with T1DM; 2544 adults with long-term T1DM (DM duration: 16.2 years)	General Finnish population; 6655 control subjects without diabetes	10 years 14 years		SMR 2.58 [95% CI 2.07–3.18]; SMR 1.33 (95% CI 1.06–1.66); IHD mortality 4.34 (95% CI 2.49–7.57)
Swedish National Diabetes Register (NDR) [8]	27,195 T1DM patients;	135,178 matched controls selected from the general population in Sweden	10 years	Hazard ratio (HR) (95% CI): 11.44 (7.95–16.44); T1DM onset age < 10 years and 3.85 (3.05–4.87); T1DM onset age: 26–30 years)	CVD mortality HR: 7.38 (3.65−14.94); T1DM onset age <10 years) and 3.64 (2.34−5.66); T1DM onset age 26–30 years

CVD = cardiovascular disease; T1DM = Type 1 diabetes mellitus; CMR = cumulative mortality rate; CHD = coronary heart disease; CAD = coronary artery disease; SMR = standardized mortality rate; IHD = ischemic heart disease; CI = confidence interval; HR = hazard ratio; IRR = incidence rate ratio; NCG = non comparison group; M = men; W = women.

### 2.2. Preclinical or Subclinical Atherosclerosis 

The clinical presentation of atherosclerosis occurs at middle age, but the atherosclerotic process begins during childhood and is accelerated by the presence of risk factors [26,27,28]. The development of the atherosclerotic process occurs faster in children with T1DM than in non-diabetic children, which probably is related to the increased incidence of other cardiovascular risk factors, such as high blood pressure (HBP), total cholesterol, low density lipoprotein cholesterol (LDL-C), inflammatory markers, and insulin resistance [29]. In this context, there has been growing interest in methods that detect subclinical atherosclerosis as a predictive method for future CVD events and to prevent excessive morbidity and mortality due to CVD in T1DM patients [30,31]. Available data based on the assessment of intima-media thickness (IMT), computed tomography (CT) for coronary artery calcium (CAC), coronary computed tomographic angiography (CCTA), and arterial stiffness suggest that preclinical atherosclerosis is much more frequent and extensive in T1DM patients. Carotid intima-media thickness (cIMT), measured through ultrasonography, is a predictive method for CVD events used in large prospective studies on asymptomatic non-diabetic subjects older than 45 [32,33,34,35,36,37,38,39] and also has been used in studies on children, adolescents, and young adults with T1DM. These studies revealed increased cIMT in T1DM patients compared with matched control subjects, which is associated with glycemic control and other cardiovascular risk factors [40,41,42,43,44]. Femoral intima-media thickness (fIMT) has been less studied than cIMT, but also is correlated with conventional risk factors and CAD, much more than cIMT [45,46]. Studies on T1DM patients revealed greater fIMT than in non-diabetic subjects [47]. CAC score is an excellent predictor of atherosclerosis and has been noted as an independent risk factor for future coronary events in non-diabetic and T2DM patients [48]. T1DM increases the prevalence and severity of coronary calcification, with both parameters being greater in women than men. This usually is associated with diabetes duration and the presence of other CVD risk factors/markers, such as body mass index, waist circumference, intra-abdominal fat, free fatty acids, smoking, insulin resistance, physical inactivity levels, and HbA1c [49,50,51]. CCTA allows for detection of luminal stenosis, and recent multicentric studies have confirmed CCTA’s prognostic value for CVD events and CVD mortality [52]. In the international Coronary CT angiography Evaluation for Clinical Outcomes (CONFIRM) multicenter registry, DM patients (T1DM and T2DM) showed higher prevalence, extent, and severity of CAD compared with matched non-diabetic subjects. Even more importantly, at a comparable degree of CAD, DM patients had higher risk of mortality than non-diabetic subjects [53]. Endothelial function, assessed using different methods, is altered even at very early stages of T1DM [54,55]. Endothelial dysfunction score is correlated with blood glucose levels and is associated inversely with DM duration. Therefore, it is not surprising that T1DM patients who had endothelial dysfunction markers were more likely to develop CHD in the Pittsburgh EDC study [56]. A recent systematic review and meta-analysis of 90 studies [31] confirmed that T1DM patients (mean age: 23.4 ± 11.3 years; diabetes duration: 10.2 ± 5.8 years) have significantly greater cIMT (SMDs [standard mean differences]: 0.89; 95% CI, 0.69–1.09; *p* < 0.001), significantly lower endothelium-dependent flow-mediated dilation (SMD: −1.45%, 95% CI, −1.74 to −1.17, *p* < 0.001), significantly increased velocity of the carotid-femoral pulse wave (SMD: 0.57, 95% CI, 0.03–1.11, *p* < 0.001), and a significant decrease in dilation mediated by glyceryl trinitrate (SMD: −1.11, 95% CI, −1, 55 to −0.66; *p* < 0.001) than controls.

Overall, preclinical CVD is more common and more extensive in T1DM patients, even at early disease stages, and even though some data suggest that preclinical CVD can be a predictive factor for future CVD events, whether application of these methods could improve T1DM management and reduce the diabetes-related burden of CVD is unclear.

### 2.3. CVD Risk Factors in T1DM Patients

The pathophysiology underlying cardiovascular events in T1DM patients remains unclear, and conventional CVD risk factors’ relative role in T1DM is not well-defined. Conventional risk factors—such as hyperlipemia, HBP, and smoking—are related to CVD in T1DM patients [57,58]. However, in contrast to what usually happens with T2DM patients, these risk factors are rarely present when T1DM diagnosis occurs and appear some years after a diabetes diagnosis. In fact, the Framingham model in the Pittsburgh EDC study underestimates the prediction of cardiovascular events in T1DM because conventional CVR factors cannot explain increased CVR in T1DM patients, suggesting the presence of specific risk factors for these subjects [59]. For instance, T1DM patients have long-term hyperglycemia that is unrelated to other associated metabolic disorders compared with T2DM patients. Below, we review the specific risk factors in the CVD pathophysiology, as well as those factors that are relevant to the evaluation and management of T1DM patients.

#### 2.3.1. Hyperglycemia

Pathophysiologic, epidemiologic, and interventional studies have found that long-term hyperglycemia is the major etiopathogenic factor for atherosclerosis in T1DM patients. Hyperglycemia is associated with the development of microvascular complications, such as nephropathy or neuropathy, which are risk factors for CVD [60,61,62,63]. These complications could be related to multiple processes activated by hyperglycemia, such as oxidative stress, protein kinase C activation, and non-enzymatic glycosylation. These processes also favor atherosclerosis development [64,65]. 

As previously mentioned, hyperglycemia is related to preclinical atherosclerosis [43,44] and the extent of atherosclerosis is correlated to HbA1c, as demonstrated by the Oslo Study over 18 years of follow-up. This study demonstrated that a 1% increase in mean HbA1c is associated with a 6.4% increase in coronary vessel stenosis [66]. However, epidemiologic evidence relating hyperglycemia to the clinical events of coronary cardiopathy are not homogeneous in T1DM patients [60,67,68,69,70]. In the EURODIAB [60], Pittsburgh EDC [67,68], and WESDR [70] studies, no association was found between glycemic control and CVD, although in the last two studies, a correlation was found between glycemic control and cardiovascular mortality. In the Swedish register-based observational study, the HR for major CHD was 1.3 in T1DM patients with a 1% increase in HbA1c [69], and T1DM patients with a HbA1c of 6.9% or lower still have a risk of death from cardiovascular causes that is two-fold higher than that of the general population. The risk of death from cardiovascular causes in T1DM patients with HbA1c ≤ 6.9% is twice as high as those in the general population, while the risk for those with very poor glycemic control (HbA1c ≥ 9.7%) is 10 times higher [22]. Data from the DCCT/EDIC study provided definitive findings on the beneficial effects from better glycemic control on CVD events in T1DM patients. In the DCCT Study, the CV event rate was very low, and patients in intensive therapy experienced fewer CVD events than the conventional-treatment group, but without statistically significant differences. After completion of the DCCT, 93% of subjects agreed to participate in the EDIC follow-up study, in which differences in HbA1c disappeared. During a follow-up of 17 years, compared with conventional therapy, intensive treatment reduced the risk of any cardiovascular event by 42 percent; the risk of nonfatal myocardial infarction, stroke, or death from CVD by 57 percent; finally, the combined endpoint of stroke, nonfatal myocardial infarction, or CVD death increased by 57 percent [71,72]. These outcomes are explained mainly by differences in HbA1c during DCCT and support the implication of the “metabolic memory” process among long-term benefits from optimal glycemic control for CVD. The potential mechanisms of this “metabolic memory” include a greater formation of cellular oxygen-reactive species and advanced glycation products as a response to chronic hyperglycemia, which would activate the mechanisms involved in the pathogenesis of T1DM-related complications [73,74,75]. Finally, glycemic control optimization also reduces other factors related to increased cardiovascular risk, such as various manifestations of renal disease (microalbuminuria or macroalbuminuria and/or impaired glomerular filtration) [76,77] and quantitative and qualitative lipid disorders [78,79,80]. 

#### 2.3.2. Hypoglycemia and Glucose Variability

In experimental conditions, acute hypoglycemia has adverse cardiovascular effects that include subclinical inflammation, endothelial dysfunction, thrombosis, ischemia, autonomic dysfunction, and arrhythmia––effects that can promote atherothrombosis, cardiac ischemia, and abnormal cardiac repolarization [81,82,83]. Although these mechanisms’ clinical relevance in the incidence of CV events still needs to be established, and clinical acute hypoglycemia rarely is associated with cardiovascular events, a recent study of T1DM patients suggested that recurrent hypoglycemia can promote premature development of CVD [84]. In fact, symptomatic and asymptomatic hypoglycemia are related to cardiac arrhythmias and increased mortality in T2DM patients [85,86]. Therefore, in the absence of new data, it is reasonable to establish individual and adjusted glycemic objectives to avoid induced arrhythmias from potentially fatal hypoglycemia, especially in subjects with CVD and/or increased cardiovascular risk. 

Increased glycemic variability has been found to be an independent cardiovascular risk factor in subjects without diabetes and with T2DM [87,88,89]. An association between glycemic variability and CAC scores has been reported in men with T1DM in the Coronary Artery Calcification in Type 1 Diabetes study [90], and mechanistic data have suggested that glucose variability could play a role in CVD through non-enzymatic glycation, oxidative stress, activation of inflammatory pathways, and endothelial dysfunction [91,92,93,94,95]. However, at present, no study has investigated the relationship between glycemic variability and CVD in T1DM, and the effect from glycemic variability on the development of CVD requires future confirmation. 

#### 2.3.3. Insulin Resistance and Metabolic Syndrome

Patients with T1DM usually present low BMI and late risk of HBP and dyslipidemia. Although there is a generalized idea that patients with T1DM have normal BMI and a late risk of BPH and dyslipidemia, especially when compared with patients with T2DM, the truth is that the prevalence of obesity and metabolic syndrome is increased in T1DM over the last years to values comparable to those of the general population [96,97,98]. This increase in the prevalence of obesity and metabolic syndrome could be related to intensive glycemic control issues corresponding with the DCCT study, which demonstrated that intensive therapy was correlated with excessive weight gain, impaired lipid profile, HBP, obesity, insulin resistance, and inflammation [99]. Abdominal obesity is a central component of metabolic syndrome and in T1DM, it is also independently associated with cardiovascular complications, marked impaired glycemic control, low-grade inflammation, HBP, and hypertriglyceridemia [100] Also, lean T1DM patients present more insulin resistance than matched control subjects [101].These findings are relevant considering that metabolic syndrome is associated with an additional increase in CVD and diabetes-related mortality after adjustment for traditional confounders and diabetic nephropathy [102]. Furthermore, metabolic syndrome also limits intensive therapy benefits on CVR reduction in T1DM patients. 

#### 2.3.4. Dyslipidemia

It is common for poorly controlled T1DM patients to have higher LDL-C and lower HDL-C levels than healthy subjects, whereas lipid levels from well-controlled T1DM patients are similar to those of individuals without diabetes [78,103]. Although the negative effect from diabetes on lipid levels is more evident in women than in men, the optimization of glycemic control normalizes or improves lipid values in both men and women. Regarding LDL-C, glycemic optimization reduces the proportion of patients in poor glycemic control, with LDL-C > 100 mg/dL and >130 mg/dL from 78% and 42% to 66% and 26%, respectively [78]. These data strongly indicate that a significant number of patients could need additional lipid-lowering intervention. However, LDL-C’s contribution to CVR in T1DM patients has elicited conflicting research results. On one hand, the Swedish National Diabetes Register suggests that LDL-C does not appear to be a good marker of cardiovascular risk in primary prevention in T1DM patients [104]. However, LDL-C appeared to be an independent risk factor for CVD or Major adverse cardiovascular events (MACE) in the DCCT/EDIC study [105]. Furthermore, the Cholesterol Treatment Trialists (CTTs) Collaboration’s meta-analysis found that every 1 mmol/L decrease in LDL-C induced a 21% relative risk reduction in MACE in T1DM patients [106]. 

T1DM patients, even those with good glycemic control, also present qualitative alterations in lipoproteins associated with increased atherogenic potential [79,107]. Although the effect on development of CVD in T1DM patients has not been established clearly, some types of modified LDL have been associated with the presence and progression of atherosclerosis in T1DM patients [107]. Low HDL-C is the most frequent dyslipidemia-related disorder in poorly controlled subjects with T1DM [78], and some qualitative alterations have been reported in HDL from T1DM, including abnormalities in composition [108] and decreased antioxidant action and cholesterol efflux capacity [109,110], which are likely involved in the enhanced development of atherosclerosis observed in T1DM patients.

#### 2.3.5. High Blood Pressure

HBP is frequent in T1DM patients. In the Coronary Artery Calcification in Type 1 Diabetes (CACTI) Study, hypertension was much more common in T1DM patients than in gender- and age-matched control subjects (43% vs. 15%, *p* < 0.001) [111]. In the EURODIAB IDDM Complications Study, approximately one quarter of patients showed blood pressure > 140/90 mmHg or received therapy for hypertension [112]. In addition to nephropathy and obesity, hyperglycemia also may contribute to HBP over the long term. In the DCCT/EDIC study, higher HbA1c was associated strongly with increased risk of HBP, and intensive hypoglycemic therapy reduced the risk of HBP by 24% [113]. 

#### 2.3.6. Diabetic Kidney Disease 

Kidney disease—present as microalbuminuria, an impaired glomerular filtration rate (GFR) or both—is a complication of T1DM that is associated strongly with CVD in DM. In fact, the presence of diabetic nephropathy dramatically increases the risk of CHD. Therefore, after 20 years of disease duration, about 29% of patients with childhood diabetes and nephropathy will present future CAD, compared with 2–3% of similar patients without diabetic kidney disease (DKD) [114]. In a recent review, T1DM and microalbuminuria patients revealed a relative risk (RR) of 2.1 (95% CI, 1.2–3.5) for CHD mortality and 2.0 for CVD (95%IC, 1.5–2.6) [115]. The risk of all-cause mortality increases with the severity of chronic kidney disease [116,117]. The overlap of DKD and CVD risk factors—such as hyperglycemia, HBP, obesity, and insulin resistance—has been proposed as a potential explanation for the increased risk of CVD associated with DKD in T1DM patients. Therefore, DKD will determine CVD risk severity and duration. Other potential factors also exist, but probably the most relevant is DKD’s contribution to worsening other traditional CVD risk factors, such as HBP, dyslipidemia, and insulin resistance.

### 2.4. Assessment of CVR in T1DM Patients

International guidelines on CVD prevention recommend lipid-lowering, blood pressure-lowering, and glucose-lowering treatments to achieve respective targets, and people with higher individual CVR will benefit more in absolute terms from preventive treatment. Therefore, risk estimation in type 1 diabetes could help individualize treatments, but current guidelines emphasize that decisions to treat and the intensity of treatment should be based on assessments of the patient’s next 10 years. Most CVD prevention guidelines’ recommendations on T1DM are derived from T2DM studies, despite known risk factors seemingly operating differently in T1DM, and that in young people with T1DM, a low absolute risk may conceal a very high relative risk that requires intervention [10]. Age appears to be by far the most significant factor, followed by time-weighted mean HbA1c, with other traditional CVD risk factors (blood pressure, LDL-C) making a demonstrable, independent contribution only after 15 to 20 years from diagnosis [105,118]. Even at <40 years of age, men and women with T1DM have a respective 5- and 10-fold relative risk of experiencing a coronary heart disease (CHD) event, and the risk remains higher with a younger onset age [8]. Hyperglycemia appears to exert a more profound effect on cardiovascular risk in T1DM than T2DM patients [22,71]. Accordingly, risk calculator engines available for the general population and for T2DM patients—such as Framingham and the UK Prospective Diabetes Study [UKPDS] models, which do not account for these factors or the presence of microvascular complications—underestimate CVD risk in T1DM patients [59]. In the Pittsburgh EDC Study, both the Framingham equations and the UKPDS Risk Engine predicted the risk of a CHD event in T1DM patients poorly. Observed and expected probabilities differed significantly for both hard and total CHD outcomes, with the largest discrepancies in the highest-risk categories [59]. Among the few risk scores developed for CVD prediction in T1DM [119,120,121], the Steno Type 1 Risk Score is probably the most promising score for predicting the first fatal or non-fatal CVD event among the T1DM population. As opposed to other models, the Steno Type 1 Risk Engine includes 10 risk factors (age, gender, diabetes duration, HbA1c, systolic blood pressure (BP), LDL-C, glomerular filtration rate, albuminuria, smoking, and exercise) and demonstrated a strong performance in predicting 10-year CVD events in a cohort of 4306 T1DM adults without previous CVD events who used an outpatient clinic at the Steno Diabetes Center in Denmark [120]. The Steno Risk Engine was superior in predicting five-year risk of fatal/nonfatal atherosclerotic cardiovascular disease (ASCVD) in the Steno cohort compared with the Swedish T1DM Risk Score, UKPDS Risk Engine, and Pooled Cohort risk equation. Moreover, it was able to identify individuals with subclinical atherosclerosis in Spain and Italy’s adult T1DM population [122,123]. However, despite the rather small number of events, absolute risk was overestimated significantly in Italian T1DM patients, considering that the observed event rate was significantly lower than the estimated one (3 vs. 13; 95% CI 12–14; *p* < 0.001) [124], and given that early onset T1DM is one of the major determinants of CVD risk events, the low representability of T1DM patients with a diagnosis in early childhood is a limitation. Lifetime risks and benefits from cardiovascular preventive interventions are another approach that could be useful in younger T1DM patients. Patients below age 40 have less risk of developing a cardiovascular event over a 10-year period, but their lifetime risk of developing cardiovascular disease is very high. Thus, estimating overall lifetime risk may be a better strategy for identifying patients who are at high risk earlier in life, which can allow for early intervention and prevent atherosclerosis development. Although models have been developed for patients with and without diabetes [125,126], and lifetime risk is touched on in the 2019 European Society of Cardiology (ESC) guidelines [127], shortcomings in these predictive models remain. 

Using cardiovascular imaging for risk assessment of atherosclerotic CVD in asymptomatic patients is controversial. According to the 2019 ESC/European Atherosclerosis Society (EAS) guidelines, CAC scoring, carotid plaque, and abnormal ankle-brachial index (ABI) may be viewed as potentially useful in assessing risk in asymptomatic patients with intermediate or borderline cardiovascular risk, and CCTA may be useful in screening for CAD in asymptomatic patients with diabetes [127]. The American Diabetes Association (ADA) does not recommend screening asymptomatic patients because the ultimate balance between benefits, costs, and risks of such an approach in asymptomatic patients remains controversial, particularly in the modern setting of aggressive ASCVD risk factor control [128]. Routine assessment of circulating biomarkers is not recommended for CVR stratification [129].

The 2019 ESC/EAS guidelines classify T1DM patients into very high CVD risk (≥10% 10-year risk of fatal CVD events), high risk (5–9% 10-year risk of fatal CVD), and moderate risk (3–4% 10-year risk of fatal CVD events) (Table 2) using essentially observational studies for stratification, and they are never validated in relation to measured CVD events. According to ESC/EAS criteria, most T1DM patients are at high/very high risk, which implies very ambitious targets for LDL-C, more stringent blood pressure control, and the use of antiplatelet agents [127]. Recently, Tecce et al. [130] evaluated the concordance between 2019 ESC/EAS CVD risk classification and 10-year CVD risk predictions, according to the Steno Type 1 Risk Engine, in 575 adults with T1DM with a mean age of 36 ± 12 years. Using ESC/EAS criteria, a large proportion (45%) of T1DM patients without CVD are classified at very high CVD risk. However, among them, none who were <35 years old and only 12% of those ≥35 years old could be confirmed to be at very high CVD risk by the Steno Type 1 Risk Engine predicting algorithm. This study highlights the great difference for initiation and eligibility of statin and other lipid-lowering therapies according to the tool used for risk stratification, thereby suggesting a major impact on the clinical care of these patients, particularly the younger ones. Pending studies that validate predictive CVD risk algorithms vs. event rates and that identify those individuals for whom aggressive cardio-protective treatment is efficient, current recommendations [127,128,129] should be applied by: considering CVR stratification using a risk engine calculator available to this population; clinical judgment that considers age, history of glycemic control, and the coexistence of genetic dyslipidemia or other factors or conditions associated with increased CVR; and the patient’s opinion.

### 2.5. CVD Prevention in T1DM Patients

Few trials have been designed specifically to assess CVR reduction strategies’ impact on T1DM patients; thus, despite important pathophysiology differences and that known risk factors seem to operate differently in T1DM patients, most of the evidence supporting lifestyle changes and pharmacological interventions to reduce cardiovascular risk comes from trials of patients without diabetes or with T2DM. 

Abundant and consistent epidemiological and experimental evidence indicates that hyperglycemia is associated with and accelerates the atherosclerotic process. In addition, DCCT and UKPDS studies that enrolled relatively young T1DM adults and newly diagnosed T2DM patients, respectively, found reductions in CVD after prolonged follow-up, despite the fact that the initial difference in glycemic control was not maintained during follow-up [131,132]. A recent meta-analysis indicates that intensive glycemic control decreases long-term incidence of major cardiovascular events, especially in patients with a diabetes duration <10 years at baseline, without prevalent CVD, and when the follow-up is >10 years [133]. As previously indicated, hypoglycemia and glucose variability also could play a role in the enhanced development of atherosclerosis observed in T1DM patients. Thus, maintaining optimal glycemic control in the long term is mandatory to decrease the probability of having a macrovascular event in patients with short diabetes duration, no previous cardiovascular events, and long life expectancy. A target of achieving an HbA1C < 7% (53 mmol/mol) without significant risk of hypoglycemia is appropriate for many T1DM patients, but must be individualized and, whenever possible, used in conjunction with metrics of continuous glucose monitoring, including time in range of > 70% and time below range < 4% [134,135]. Most T1DM patients should be treated with intensive insulin regimens, either via multiple daily injections or continuous subcutaneous insulin infusion, and they should self-monitor glucose levels up to 6–10 times daily using a glucose meter or intermittently scanned/real-time continuous glucose monitoring [136]. 

CVD risk remains significantly high even in well-controlled T1DM patients, indicating that other potential factors are involved, and evidence indicates that younger onset is associated with greater loss of life expectancy. Thus, longer-term benefits from more aggressive management of CVD risk factors may be possible in those who develop diabetes under age 20. However, the use of cardiovascular medications, such as angiotensin-converting enzyme inhibitors (ACEIs) and statins, is very low in T1DM children and adolescents with CVD risk factors [137]. This probably reflects the absence of randomized controlled trials in T1DM patients and that, unlike subjects with T2DM, adults and adolescents with T1DM do not usually display the metabolic syndrome components associated with insulin resistance.

As occurs in general and T2DM populations, dyslipidemia should be treated with statins to reduce CVD in T1DM patients. Although lipid and lipoprotein levels are often within the normal range in T1DM patients, LDL-C is a significant predictor of cardiovascular events and mortality in T1DM patients [105,138,139], and even T1DM patients with good glycemic control manifest several alterations in lipoprotein composition and functionality that may be important to CVD risk in T1DM patients. Statin therapy, the mainstay of treatment for lowering LDL levels, has been shown to reduce mortality and cardiovascular events. In the Heart Protection Study (lower age limit: 40), the subgroup of 600 T1DM patients had a reduction in risk that was proportionately similar (although insignificant statistically) to T2DM patients [140]. A meta-analysis that included 18,686 patients with DM (1466 T1DM patients, mean age 55, and 56% with previous CVD events) demonstrated that a statin-induced reduction in LDL-C by 1.0 mmol/L (40 mg/dL) was associated with a 9% reduction in all-cause mortality and a 21% reduction in the incidence of major CV events [106]. Similar benefits were seen in both T1DM and T2DM patients. In a prospective cohort study in adults with T1DM who had no underlying CVD, lipid-lowering therapy was associated with decreased all-cause mortality [141], and in the AdDIT trial in adolescents with T1DM, treatment with statin therapy for 2–4 years reduced LDL-C concentrations, but did not improve carotid intima–media thickness [142]. Unfortunately, no data are available on the efficiency and age at which statin therapy should be initiated in young T1DM patients. 

Lipid-lowering drugs—such as ezetimibe, proprotein convertase subtilisin/kexin type 9 (PCSK9) inhibitors, and Icosapent ethyl—have been found to elicit cardiovascular risk reduction in T2DM patients, but data on T1DM patients are currently lacking. Recent studies have demonstrated that T1DM patients have increased cholesterol absorption [143], and ezetimibe was more effective in lowering LDL for T1DM vs. T2DM patients [144], suggesting that ezetimibe may be particularly effective in T1DM patients. Finally, youths with T1DM have increased PCSK9 concentrations [145], and PCSK9 inhibitors reduce LDL-C levels by 47.8% vs. placebo in T1DM patients [146]. 

Due to the absence of randomized controlled trials with T1DM patients, most guidelines fail to distinguish T1DM from T2DM patients. Current ADA and ESC/EAS recommendations for lipid modification in T1DM patients are provided in Table 2 and Table 3 [127,128,147]. ADA guidelines are less aggressive, recommending statin therapy for persistent LDL-C > 160 mg/dL unless other CVD risk factors are present in children and adolescents, while in the ESC/EAS Guidelines, statin therapy may be considered in T1DM patients ages ≤ 30, with evidence of end organ damage and/or an LDL-C > 100 mg/dL (2.5 mmol/L). The ADA Standards of Medical Care in Diabetes 2021 do not consider T1DM duration, whereas the ESC/EAS Guidelines place special emphasis on T1DM diabetes duration as a CVD risk factor, viewing those with a T1DM duration of ≥10 years as high-risk, with an LDL-C goal < 70 mg/dL, and those with a T1DM duration of ≥20 years as very-high-risk, with an LDL-C goal < 55 mg/dL (Table 2). ADA and ESC/EAS recommendations consider the addition of icosapent ethyl to reduce cardiovascular risk in patients with ASCVD or other cardiovascular risk factors on a statin with controlled LDL-C, but with triglycerides 135–499 mg/dL [128], and ESC/EAS also considers fenofibrate or bezafibrate in combination with statins in high-risk patients with an LDL-C goal, with TG levels > 200 mg/dL (>2.3 mmol/L).

The ADA [128] recommends that most patients with diabetes and hypertension get treated with an eye toward a systolic BP goal of <140 mmHg and a diastolic BP goal of <90 mmHg, while for individuals at high risk of CVD, a blood pressure target of 130/80 mmHg may be appropriate if it can be attained safely. For children and adolescents, the blood pressure goal is less than the 90th percentile for age, height, and gender for patients younger than 13, or less than 130/80 mm Hg for those ages 13 and up. In 2019, the ESC/EAS Guidelines [129] recommended treatment with antihypertensive drugs for those with diabetes and a BP > 140/90 mmHg, with therapeutic targets of systolic BP ≤ 130 mmHg and diastolic BP < 80 mmHg. Pharmacological treatment should include ACE inhibitors or angiotensin receptor blockers, but frequently, a multi-pronged therapeutic approach is required for optimum results. The ADA recommends the use of aspirin (75–162 mg/day) in secondary prevention and also recommends using them to reduce high cardiovascular risk (ages > 50 years and at ≥ 1 major risk factor or chronic kidney disease/albuminuria) in those with low bleeding risk. 

## 3. Conclusions

T1DM is associated with an almost threefold higher mortality rate than that of the general population, and CVD is a major cause of this mortality, but the underlying mechanisms remain poorly understood. Although hyperglycemia plays an important role, CVD risk remains high, even in well-controlled T1DM patients, indicating that other factors—such as glucose variability, hypoglycemia, and qualitative and functional abnormalities of lipoproteins—could be involved. Lifestyle interventions and optimal glycemic control without significant hypoglycemia are mandatory to reduce CVR in T1DM patients, but there are concerns about CVR stratification and the use of statins and antihypertensive drugs in this population, especially in young T1DM patients. Further research is needed to clarify the factors involved in premature CVD in T1DM patients, including gender and ethnic differences, to better predict and stratify CVR, and to elucidate the efficiency and age at which current cardiovascular medications should be initiated in young T1DM patients, and identify potential pharmacological targets for novel therapeutic approaches to prevent the development and progression of atherosclerosis in T1DM patients.

## Figures and Tables

**Table 2:** Cardiovascular risk (CVR) categories, LDL-C targets, and lipid-lowering therapy in diabetes patients—2019 ESC/EAS Guidelines.

Cardiovascular Risk Categories		LDL-C Target	Lipid-Lowering Therapy
Very high risk	Patients with diabetes and documented ASCVD, either clinical, or unequivocal on imaging, or other target organ damage, ^a^ or three or more major risk factors, or early onset T1DM of long duration (>20 years)	-LDL-C < 1.4 mmol/L (< 55 mg/dL) and LDL-c reduction ≥ 50% -IF two ASCVD events within two years, consider LDL-C < 1.0 mmol/L (40 mg/dL).	-If LDL-C is above the targeted goal, recommend statin therapy. -If the goal is not achieved with maximum tolerated statin therapy, a combination with ezetimibe is recommended. -If ASCVD and LDL-C are above the targeted goal, consider adding a PCSK9 inhibitor. -If ASCVD and LDL-C have not reached the targeted goal, recommend adding a PCSK9 inhibitor.
High Risk	Patients with DM without target organ damage, ^a^ with DM duration ≥10 years or another additional risk factor.	LDL-c < 1.8 mmol/L (<70 mg/dL) and LDL-c reduction ≥ 50%	-If LDL-C is above the targeted goal, recommend statin therapy. -If the goal is not achieved with maximum tolerated statin therapy, a combination with ezetimibe is recommended.
Moderate risk	Young patients (T1DM <35 years or T2DM <50 years) with DM duration <10 years, without other risk factors	LDL-c < 2.6 mmol/L (<100 mg/dL)	Statin therapy should be considered if LDL-C rises above the targeted goal.

ASCVD = atherosclerotic cardiovascular disease; LDL-C = low-density lipoprotein cholesterol; SCORE = Systematic Coronary Risk Estimation; DM = diabetes mellitus; T1DM = type 1 DM; T2DM = type 2 DM; ^a^ Target organ damage is defined as microalbuminuria, retinopathy, or neuropathy. Secondary goals: Non-HDL-C <2.2, 2.6, and 3.4 mmol/L (<85, 100, and 130 mg/dL) for very high-, high-, and moderate-risk, respectively. ApoB < 65, 80, and 100 mg/dL for very high-, high, and moderate-risk, respectively.

**Table 3:** 2021 ADA guidelines for lipid management in patients with diabetes.

Primary Prevention	
Age	Recommendations
Children and adolescents	>10 years, consider the use of statins if LDL-C > 160 mg/dL (4.1 mmol/L) or LDL cholesterol > 130 mg/dL (3.4 mmol/L) and one or more cardiovascular disease risk factors are present. LDL-C goal: <100 mg/dL (2.6 mmol/L).
20–39 years	If additional ASCVD is present, consider the use of statin therapy. LDL-C goal: <100 mg/dL (2.6 mmol/L)
40–75 years	Moderate-intensity statin therapy LDL-C goal: <100 mg/dL (2.6 mmol/L)
	If multiple ASCVD is present, or the patient is 50 to 70 years old, it would be reasonable to use high-intensity statin therapy. LDL-C goal: <100 mg/dL (2.6 mmol/L)
	With a 10-year ASCVD risk of 20% or higher, it would be reasonable to add ezetimibe to maximally tolerated statin therapy to reduce LDL cholesterol levels by 50% or more.
>75 years	If on statin therapy, it is reasonable to continue.
	It may be reasonable to initiate statin therapy after considering potential benefits and risks.
**Secondary Prevention**	
All ages with ASCVD	**Recommendations**
	High-intensity statin therapy
	If the risk is very high using specific criteria, and LDL-C ≥ 70 mg/dL after a maximally tolerated statin dose, consider adding ezetimibe or a PCSK9 inhibitor to help lower LDL-C. LDL-C goal: <70 mg/dL (1.8 mmol/L)

Moderate-intensity statin therapy (lowers LDL-C by 30–49%): atorvastatin 10–20 mg, rosuvastatin 5–10 mg, simvastatin 20–40 mg, lovastatin 40 mg, fluvastatin XL 80 mg, pitavastatin 1–4 mg or pravastatin 40–80 mg/day; high-intensity statin therapy (lowers LDL cholesterol by >50%): atorvastatin 40–80 mg and rosuvastatin 20–40 mg. ASCVD = atherosclerotic cardiovascular disease; LDL-C = low-density lipoprotein cholesterol.
